# A single-plasmid-based, easily curable CRISPR/Cas9 system for rapid, iterative genome editing in *Pseudomonas putida* KT2440

**DOI:** 10.1186/s12934-024-02634-4

**Published:** 2024-12-30

**Authors:** Qifeng Wen, JinJin Chen, Jin Li, Ida Putu Wiweka Dharmasiddhi, Maohua Yang, Jianmin Xing, Yilan Liu

**Affiliations:** 1 State Key Laboratory of Petroleum Molecular & Process Engineering, Institute of Process Engineering, Chinese Academy of Sciences, Beijing, China; 2 College of Chemical Engineering, University of Chinese Academy of Sciences, Beijing, China; 3 Department of Chemical Engineering, University of Waterloo, Waterloo, Canada

**Keywords:** *Pseudomonas putida* KT2440, All-in-one CRISPR/Cas9 system, Plasmid curing, Genome editing, Biosynthesis

## Abstract

**Background:**

*Pseudomonas putida* KT2440, a non-pathogenic soil bacterium, is a key platform strain in synthetic biology and industrial applications due to its robustness and metabolic versatility. Various systems have been developed for genome editing in *P. putida*, including transposon modules, integrative plasmids, recombineering systems, and CRISPR/Cas systems. However, rapid iterative genome editing is limited by complex and lengthy processes.

**Results:**

We discovered that the pBBR1MCS2 plasmid carrying the CRISPR/Cas9 module could be easily cured in *P. putida* KT2440 at 30 ^o^C. We then developed an all-in-one CRISPR/Cas9 system for *yqhD* and *ech-vdh-fcs* deletions, respectively, and further optimized the editing efficiency by varying homology arm lengths and target sites. Sequential gene deletions of *vdh* and *vanAB* were carried out rapidly using single-round processing and easy plasmid curing. This system’s user-friendliness was validated by 3 researchers from two labs for 9 deletions, 3 substitutions, and 2 insertions. Finally, iterative genome editing was used to engineer *P. putida* for valencene biosynthesis, achieving a 10-fold increase in yield.

**Conclusions:**

We developed and applied a rapid all-in-one plasmid CRISPR/Cas9 system for genome editing in *P. putida*. This system requires less than 1.5 days for one edit due to simplified plasmid construction, electroporation and curing processes, thus accelerating the cycle of genome editing. To our knowledge, this is the fastest iterative genome editing system for *P. putida*. Using this system, we rapidly engineered *P. putida* for valencene biosynthesis for the first time, showcasing the system’s potential for expanding biotechnological applications.

**Supplementary Information:**

The online version contains supplementary material available at 10.1186/s12934-024-02634-4.

## Introduction

*Pseudomonas putida* KT2440, a non-pathogenic soil bacterium, excels as a model organism in synthetic biology and a valuable candidate for diverse industrial uses. Known for its safety, robustness, solvent tolerance, and metabolic flexibility, it processes diverse organic compounds, supporting sustainable waste management and complex molecule synthesis for green chemistry and circular economies [1, 2]. With genetic modifications, *P. putida* is tailored to produce valuable biochemicals like bioplastics, pharmaceuticals, and fine chemicals [3]. This positions it as a key player in biotechnology, offering sustainable alternatives to traditional chemical synthesis and enhancing industrial sustainability.

Genome editing is crucial for integrating expression modules into chromosomes, ensuring stable inheritance, consistent production, and tighter transcriptional control in engineered bacteria for industrial use [4]. A detailed review of genome editing methods that have been developed for *P. putida* since the 1990s, including the use of transposon modules, integrative plasmids, recombineering systems, and CRISPR/Cas (clustered regularly interspaced short palindromic repeats CRISPR-associated proteins) modules [5]. Tn5 and Tn7 transposons are commonly used, with Tn5 offering flexibility in inserting sites and Tn7 providing targeted integration at specific loci like the attTn7 site [6, 7]. Integrative plasmids like pK18mobsacB employ suicide vectors for targeted gene deletions via homologous recombination [8]. Recombineering systems employ linear DNA fragments and recombinase proteins, such as λRed and Ssr, to introduce mutations, deletions, or insertions directly into the chromosome, enhancing the precision and flexibility of genomic modifications [9, 10]. In addition, site-specific recombinases, such as Cre and λ integrase, are utilized for precise genome editing [11]. These strategies allow for genomic editing in *P. putida*, though they may require prior insertion of recognition sites or have low efficiencies, posing technical challenges.

In recent years, the relative simplicity and efficiency of CRISPR/Cas9 system have catalyzed advances in genome editing across various organisms. However, the scientific community predominantly relies on previously discussed traditional methods for genome editing in *P. putida* [5]. Despite their benefits, novel CRISPR/Cas systems have struggled to transition from development to widespread applications in *P. putida*, largely due to the complexity or inefficiency of current methods, especially for iterative genome editing. For instance, CRISPR/Cas9 system was used together with λRed [12, 13] or Ssr recombinases [14] to achieve high genome editing efficiency in *P. putida*. However, these systems required multiple plasmids and two rounds of competent cell preparation, transformation, and selection, which took at least 4–5 days for a single editing. Moreover, the need to induce recombinase expression and trigger plasmid curing added further complexity to the process. The one-plasmid system has also been reported [15, 16], but the low efficiency of chromosomal integration of the λRed/Cas9n or suicide plasmid, along with the requirement for inducers, made the process still complicated and time-consuming. Given the limitations of current genome editing methods, there’s a crucial demand for simpler and faster techniques in *P. putida*. The single-plasmid-based, all-in-one CRISPR/Cas system presents itself as a promising solution for this challenge. For example, a highly efficient all-in-one CRISPR/Cas9 system within *Bacillus subtilis* was developed, but this system relies on inducers to regulate *Cas9* expression and self-targeting sgRNA for rapid plasmid curing [17]. Similarly, an all-in-one CRISPR/Cas9 system in *Acidithiobacillus ferridurans* was reported, though the plasmid curing process was time-consuming [18].

In this study, we engineered a single-plasmid-based CRISPR/Cas9 system for rapid genome editing in *P. putida* KT2440. Our system facilitates accelerated editing through a simplified single-round process and straightforward plasmid curing, without the need for an inducer. Initially, we discovered that the pBBR1MCS2 plasmid carrying the Cas9 gene could be easily cured in *P. putida* at 30 ^o^C. We then developed an all-in-one CRISPR/Cas9 system and optimized it for efficient genome editing, achieving various gene deletions, substitutions, and insertion. This system’s user-friendliness was validated by three graduate students from two different labs. As proof of concept, this system was used for iterative genome editing to synthesize valencene, resulting in a 10-fold increase of production in *P. putida* KT2440.

## Materials and methods

### Strains, culture conditions and reagents

All strains used in this study are listed in Table 1. *E. coli* DH5α and Top10 were used as the host for all cloning experiments and were grown at 37 ^o^C in Luria–Bertani liquid medium (LB; 10 g /L tryptone, 5 g /L yeast extract, 10 g /L NaCl) and solid medium (LB with 15 g /L agar). *P. putida* KT2440 strains were used for plasmid curing tests, genetic engineering, and valencene biosynthesis. They were cultured in LB media at 30 °C, unless otherwise specified. The super optimal medium with catabolic repressor (SOC; 20 g /L tryptone, 5 g /L yeast extract, 0.5 g /L NaCl, 0.186 g /L KCl, 0.95 g /L MgCl_2_ 3.6 g /L glucose) was used in the recovery step of *P. putida* competent cell transformations. Antibiotics kanamycin (Km, 50 µg /mL) and chloramphenicol (Cm, 34 µg /mL) were used when required. The standard (+)-valencene was purchased from Sigma-Aldrich and other chemicals were obtained from commercial sources.

### Plasmid construction

All plasmids and primers used in this study are listed in Table 1 and Supplementary Table S1, separately. Plasmid construction followed conventional molecular biology methodologies. Initially, linearized vector fragments were obtained through either restriction enzyme digestion or high-fidelity polymerase chain reaction (PCR). The inserted fragments were synthesized by Twist Bioscience or amplified via high-fidelity PCR, each possessing 15–20 bp overlapping sequences at their ends. Subsequently, these fragments underwent assembly and the assemble product was transferred into *E. coli* DH5α or Top10 for screening, miniprep, and whole plasmid sequencing. The modular cloning steps were shown in Supplementary Fig. S1. PCR was conducted using TSINGKE Master Mix (Tsingke Biotechnology, China), KOD-Plus -Neo (Toyobo, Japan), or KAPA2G HotStart ReadyMix kits (Kapa Biosystems, USA). Plasmid assembly was achieved using either the ClonExpress MultiS One Step Cloning Kit (Vazyme Biotech, China) or the Uni Seamless Cloning and Assembly Kit (Trans Gen Biotech, China). Purification of plasmids and DNA was carried out using Monarch kits (NEB, USA) or EZNA^®^ kits (Omega Bio-Tek, USA), following the manufacturer’s instructions.

The sgRNA design was conducted using Cas-Designer (http://www.rgenome.net/cas-designer) [19]. In brief, start by setting the PAM (Protospacer Adjacent Motif) type to SpCas9 from *Streptococcus pyogenes*, which is 5’-NGG-3’ and then select the target genome, *Pseudomonas putida* KT2440 (NC_002947.3). Next, copy and paste the target DNA sequence for potential sgRNA target searching. A list of potential sgRNA targets will be displayed, from which you should choose the sgRNA sequence with an out-of-frame score higher than 66. All sgRNA spacer sequences of the targeted gene used in this study are listed in Supplementary Table S2. We deposited a plasmid pBBR-*Cas9ΔyqhD-HA500* in Addgene (NO 220369) for reference.

**Table 1:** Strains and plasmids used in this study

Strains or plasmids	Relevant characteristics	Source
Strains		
*E. coli* DH5α	*F-φ80 lacZΔM15Δ (lacZYA-argF) U169recA1 endA1 hsdR17 (rk -*,* mk +) phoA*,* sup E44 thi − 1 gyr A96 rel A1 λ -*	TransGen Biotech
*E. coli* Top10	*F-mcrA Δ(mrr-hsdRMS-mcrBC) φ80lacZΔM15ΔlacX74recA1 araΔ139Δ(ara-leu)7697galU galK rpsL(Sm) endA1nupG*	TransGen Biotech
*P. putida* KT2440	*Wild-type strain*	In our lab
Pp1	*P. putida* KT2440 + pBBR1MCS2	This study
Pp2	*P. putida* KT2440 + pBBR-*Cas9*	This study
Pp3	*P. putida* KT2440 + pBBR-*Cas9ΔyqhD-HA500*	This study
PP*ΔyqhD*	*P. putida* KT2440 with yqhD deletion	This study
PP*Δevf*	*P. putida* KT2440 with *ech*, *vdh* and *fcs* deletions	This study
Pp*Δend*	*P. putida* KT2440 with *endAX* deletions	This study
PpV01	Pp*Δend* + pBBR-vs.	This study
PpV02	Pp*Δend* + pBBR-*vs-dxs*	This study
PpV03	Pp*Δend* + pBBR-*vs-ispA*	This study
PpV04	Pp*Δend* with *phaG* deleted + pBBR-*vs-dxs*	This study
PpV05	Pp*Δend* with *phaG* and *phaC1ZC2* deleted + pBBR-*vs-dxs*	This study
PpV06	Pp*Δend* with *phaG* and *phaC1ZC2* deleted + pBBR-*vs-dxs-ispA*	This study
PpV07	Pp*Δend* with *phaG* and *phaC1ZC2* deleted and vs. linked to *ispA in genome* + pBBR-*vs-dxs-ispA*	This study
**Plasmids**		
pBBR1MCS2	p1, pBBR replicon, mob+, Km	In our lab
pBBR-*Cas9*	p2, pBBR1MCS2 derivative, Plac and *Cas9*	This study
pBBR-*Cas9ΔyqhD-HA500*	p3, pBBR-*Cas9* derivative, sgRNA and donor DNA (500 bp per arm) of *yqhD*	This study
pBBR-*Cas9ΔyqhD-HA300*	pBBR-*Cas9* derivative, sgRNA and donor DNA (300 bp per arm) of *yqhD*	This study
pBBR-*Cas9ΔyqhD-HA150*	pBBR-*Cas9* derivative, sgRNA and donor DNA (150 bp per arm) of *yqhD*	This study
pBBR-*Cas9ΔyqhD-HA900*	pBBR-*Cas9* derivative, sgRNA and donor DNA (900 bp per arm) of *yqhD*	This study
pBBR-*Cas9ΔyqhD-HA1200*	pBBR-*Cas9* derivative, sgRNA and donor DNA (1200 bp per arm) of *yqhD*	This study
pBBR-*Cas9Δevf-HA150*	pBBR-*Cas9* derivative, sgRNA and donor DNA (150 bp per arm) of *ech-vdh-fcs*	This study
pBBR-*Cas9Δevf-HA300*	pBBR-*Cas9* derivative, sgRNA and donor DNA (300 bp per arm) of *ech-vdh-fcs*	This study
pBBR-*Cas9Δevf-HA500*	pBBR-*Cas9* derivative, sgRNA and donor DNA (500 bp per arm) of *ech-vdh-fcs*	This study
pBBR-*Cas9Δevf-HA900*	pBBR-*Cas9* derivative, sgRNA and donor DNA (900 bp per arm) of *ech-vdh-fcs*	This study
pBBR-*Cas9Δevf-HA1200*	pBBR-*Cas9* derivative, sgRNA and donor DNA (1200 bp per arm) of *ech-vdh-fcs*	This study
pBBR-*Cas9ΔcatAB*	pBBR-*Cas9* derivative, sgRNA and donor DNA of *catAB* deletion	This study
pBBR-*Cas9Δvdh*	pBBR-*Cas9* derivative, sgRNA and donor DNA of *vdh* deletion	This study
pBBR-*Cas9ΔvanAB*	pBBR-*Cas9* derivative, sgRNA and donor DNA of *vanAB* deletion	This study
pBBR-*Cas9ΔyqhD::gfp*	pBBR-*Cas9* derivative, sgRNA and donor DNA for *gfp replacing yqhD*	This study
pBBR-*Cas9ΔyqhD::rfp*	pBBR-*Cas9* derivative, sgRNA and donor DNA for r*fp replacing yqhD*	This study
pBBR-*Cas9Δ2827::MluI*	pBBR-*Cas9* derivative, sgRNA and donor DNA for *MluI* replacing *PP_2827*	This study
pBBR-*Cas9::RBS-fadH*	pBBR-*Cas9* derivative, sgRNA and donor DNA for an RBS insertion before *fadH*	This study
pBBR-*Cas9ΔendA*	pBBR-*Cas9* derivative, sgRNA and donor DNA of *endA* deletion	This study
pBBR-*Cas9ΔendX*	pBBR-*Cas9* derivative, sgRNA and donor DNA of *endX* deletion	This study
pBBR-*Cas9ΔphaG*	pBBR-*Cas9* derivative, sgRNA and donor DNA of *phaG* deletion	This study
pBBR-*Cas9ΔphaC1ZC2*	pBBR-*Cas9* derivative, sgRNA and donor DNA of *phaC1ZC2* deletion	This study
pBBR-*Cas9::vs.*	pBBR-*Cas9* derivative, sgRNA and donor DNA for vs. insertion linking with *ispA*	This study
pBBR-vs.	pBBR1MCS2 derivative, carrying vs. (valencene synthase) gene	This study
pBBR-*vs-dxs*	pBBR1MCS2 derivative, carrying *vs. and dxs* (DXP synthase) genes	This study
pBBR-*vs-ispA*	pBBR1MCS2 derivative, carrying *vs. and ispA* (GPP/FPP synthase) genes	This study
pBBR-*vs-dxs-ispA*	pBBR1MCS2 derivative, carrying vs., *dxs and ispA*	This study

### Transformation and genome editing of *P. putida*

Plasmid transformation of *P. putida* was performed *via* a modified electroporation method according to our previous report [20]. *P. putida* was grown overnight in 1 mL LB medium at 30 °C and 220 rpm. The next day, the culture was transferred into fresh LB medium at a 1:100 ratio and grown until the OD_600_ reached 0.6–0.8. Cells were collected by centrifugation at 6,000 g for 10 min, washed with ddH_2_O followed by 10% (v/v) glycerol, and resuspended in precooled 10% glycerol to achieve an OD_600_ greater than 40. Aliquots of 40 uL were distributed into 1.5 mL tubes for storage at -80 ^o^C and later use. All collection and washing steps were performed at 4 ^o^C or on an ice-water mix.

The competent cells were mixed with 50 ng plasmid DNA, incubated on ice for 5 min, and electroporated at 12.5 kV/cm for 6 ms using a Gene Pulser II (Bio-Rad, USA). The cells were then recovered in 1 mL LB at 30 °C for 60 min with shaking at 220 rpm and plated 100 uL on LB-agar with antibiotics. For genome editing, after electroporation, 1 mL of SOC medium was added to the cuvette immediately. The culture was transferred into an 8 mL tube with air-permeable cap, SOC was added to 1.5 mL, and cultured at 30 ^o^C, 140 rpm, 20 min, then 220 rpm 40 min. The cells were centrifuged at 8,000 g for 2 min and plated on LB agar with Km at 30 ^o^C overnight. Editing efficiency was determined by calculating the ratio of edited (positive) bands to the total number of bands (positive plus wild type) on an agarose gel, with the edits further verified by sequencing. Images of the agarose gel and sequencing results for all genome edits are provided in Supplementary Fig. S2. For the editing efficiency tests of *yqhD* and *ech-vdh-fcs* deletions, 20 colonies were analyzed in triplicate (60 total), and 10–20 colonies were tested for other edits.

### Plasmid curing

The plasmid curing tests were performed with *P. putida* carrying pBBR1MCS2 (Pp1) and pBBR-*Cas9* (Pp2) in LB media at 30 ^o^C, 35 ^o^C and 37 ^o^C. Additionally, *P. putida* carrying pBBR-*Cas9ΔyqhD-HA500* (Pp3) was tested together with Pp1 and Pp2 at 30 ^o^C. During culturing, 2.5 uL of culture was taken every 1–2 h and streaked on LB plate and cultured overnight at 30 ^o^C incubator. The next day, 32 single colonies were picked for colony PCR and point-to-point streak on both LB and LB Km plates to further verify the plasmid curing.

For iterative genome editing, after the first editing, the verified positive colony was cultured in 2 mL LB medium at 30 ^o^C for 8 h. Then 2.5 uL of culture was plated on a LB plate. The next day, colonies were first checked using PCR and then streaked point-to-point using a toothpick on both LB and LB-Km agar plates.

### Valencene biosynthesis and analysis

The engineered *P. putida* strains were cultured in 10 mL LB media as a seed culture. The next morning, the culture was inoculated at a ratio of 2% into fresh LB media with 10% dodecane. Fermentation in flasks for (+)-valencene biosynthesis was then carried out at 30 ^o^C and 200 rpm for 48 h. Valencene extracts in the dodecane layer were initially analyzed by gas chromatography − mass spectrometry (GC − MS) to identify compounds by comparing it with a reference standard (Fig. S3). A 30 m Rtx-5MS column (0.25 mm × 0.25 μm) was used on GC-2010 Series GC equipped with a Double-stage turbomolecular pumps (ultra) mass spectrometer. Sample aliquots of 1 µL were injected in split mode (split ratio 50:1) at 280 °C injector and 280 °C detector temperatures with a helium flow rate of 1 mL/min. An initial temperature of 40 °C was held for 1 min, 30 °C/min ramp to 160 °C (2 min), and 10 °C/min ramp to 280 °C (5 min). The ionization mode was electron impact ion source (EI), a temperature of 200 °C was set for the ion source. The total ion current was detected (TIC). Data was acquired at full-scan mode (20–800 m/z) and analyzed by using Software Xcalibur.

An Agilent GC system coupled with a flame ionization detection (GC-FID) is used to quantify valencene following a method described in previous research [21]. HP-5 column (cross-linked 5% Ph-Me siloxane; 30 m × 0.32 mm × 0.25 μm) was used on an Agilent 7890B Series GC equipped with a flame ionization detector. The temperatures of the injector and detector were set as 250 and 350 °C, respectively. Sample aliquots of 1 mL were injected in split mode (split ratio 50:1) at 250 °C injector temperature. The oven temperature program was as follows: started at 50 °C was held for 1 min, a ramp of 15 °C/min to 280 °C, and held at 280 °C for 1 min, a ramp of 20 °C/min to 300 °C, and held at 300 °C for 2 min. The valencene titters were calculated by dividing the valencene concentration of the dodecane phase by 10 because 10% (vol/vol) dodecane was added to the cell cultures.

### Statistical analysis

To assess statistical significance in this study, the means of the data sets were evaluated with a one-way analysis of variance (ANOVA) followed by the Tukey’s Honest Significant Difference (HSD) test for multiple comparisons.

## Results and discussion

### Plasmid-curing investigation

**Fig. 1:**
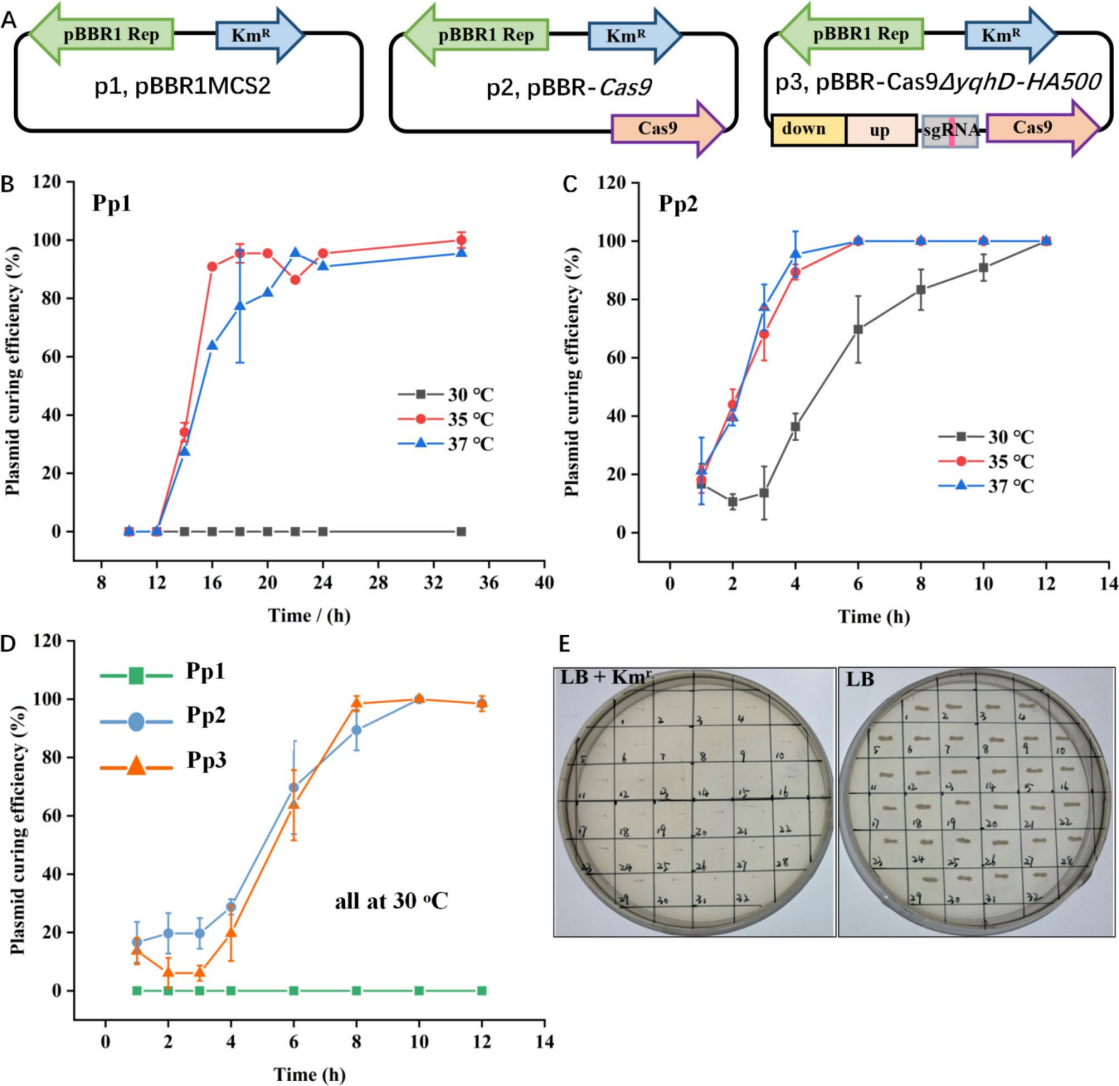
Investigation of plasmid-curing efficiencies in *P. putida* KT2440 in the absence of antibiotics. (**A**) Schematic representation of different plasmids used for stability tests. Plasmid p1 refers to pBBR1MCS2 without additional cargo; Plasmid p2 represents pBBR-*Cas9*, which carries only the *Cas9* gene; Plasmid p3 designates pBBR-*Cas9ΔyqhD-HA500*, which contains the CRISPR/Cas9 system targeting the *yqhD* gene. (**B**) Plasmid-curing tests of p1 in *P. putida* (Pp1) at 30 ^o^C, 35 ^o^C and 37 ^o^C. (**C**) Plasmid-curing tests of p2 in *P. putida* (Pp2) at 30 ^o^C, 35 ^o^C and 37 ^o^C. (**D**) Plasmid-curing tests of p1, p2, and p3 in *P. putida* (Pp1, Pp2 and Pp3) at 30 °C. (**E**) Verification of plasmid curing using point-to-point streaking. Pp1, *P. putida* carrying the plasmid p1, pBBR1MCS2; Pp2, *P. putida* carrying the plasmid p2, pBBR-*Cas9*; Pp3, *P. putida* carrying the plasmid p3, pBBR-*Cas9ΔyqhD-HA500*

Rapid plasmid-curing is important for genome editing, especially for iterative genome engineering. Due to the high copy number and intrinsic stability of modern cloning vectors, plasmid-curing can often be challenging. The broad-host-range plasmid pBBR1MCS2 features a multiple cloning site, mobilizability (RK2), and compatibility with diverse plasmid groups (IncP and IncQ), making it popular for gene expression and metabolic engineering in various bacteria, including *P. putida* [22]. However, plasmid stability can vary depending on hosts, growth conditions, selection pressure, and genetic content. For instance, the instability of pBBR-*mpd* in *Sphingomonas* sp. CDS-1 was observed [23], and the instability of pBBR-UP, a pBBR1 variant, was reported in *P. putida* [12]. The instability of the plasmid provides the possibility for rapid plasmid-curing, so we constructed a series of pBBR1MCS2 derivatives and investigated the influence of temperature changes and various CRISPR/Cas9 cargoes on pBBR1MCS2 stability in *P. putida* without antibiotics. Figure 1A depicts the loading of different cargoes onto pBBR1MCS2 before transferring into *P. putida*, resulting in strains Pp1, Pp2, and Pp3. Subsequently, we evaluated plasmid curing efficiencies in these strains.

Studies suggest that elevated culture temperatures may stress cells and decrease plasmid stability [24, 25]. Thus, to achieve rapid plasmid curing, we assessed the stability of pBBR1MCS2 at varying culture temperatures. As shown in Fig. 1B, during 34 h of continuous culture of Pp1, we found pBBR1MCS2 to remain stable in *P. putida* at 30 ^o^C, while it was eliminated within 14 h at 35 °C or 37 °C. Each experiment was performed in triplicate, ensuring that the loss of plasmids was not due to random chance. Previous studies have linked plasmid instability to specific mutations. The 3nt mutations (T-43 A, A73G and A436G) in the replication origin of pBBR1MCS2 was reported to cause its instability in *E. coli* at 42 ^o^C [26], Similarly, the poor stability with the pBBR-UP mutation (C299T) was observed [12]. Upon comparing these sequences, we confirmed that replication origin of pBBR1MCS2 in our study was identical to that of the wild type, ruling out mutations as a contributing factor to plasmid instability in our experiments.

The *Streptococcus pyogenes* (sp) CRISPR/Cas9 system, while adapted for genome editing across multiple bacteria, exhibits toxicity due to the double-strand breaks caused by SpCas9 cleavage or its tight binding to PAM sequences [27–29]. In our study, using the Plac promoter with a strong RBS sequence to solely express SpCas9 in *E. coli* DH5a consistently resulted in truncated mutations or stop codons within the *Cas9* gene, confirming its toxicity. We mitigated this by modifying the sequence upstream of the *Cas9* gene, removing spacers and intervals and replacing the RBS (Supplementary Fig. S4). This enabled successful construction of pBBR-*Cas9*, which was then transferred into *P. putida* to create strain Pp2. Figure 1C shows that in Pp2, the *Cas9* gene caused plasmid instability, with complete loss at both 35 ^o^C and 37 ^o^C within 6 h. We introduced plasmid p3 into *P. putida* to create strain Pp3. Plasmid p3 carries a complete CRISPR/Cas9 system, including *Cas9*, sgRNA, and homologous arms targeting the *yqhD* gene. Growth curves of Pp1, Pp2 and Pp3 in the presence of Km demonstrated that both Cas9 and the CRISPR/Cas9 system impact cell growth (Supplementary Fig. S5). In the absence of the antibiotic, we assessed the stability of plasmids p1, p2, and p3 in *P. putida* at 30 ^o^C, where CRISPR/Cas9 system resulted in complete plasmid elimination within 8–10 h. The plasmid curing was also confirmed by point-to-point streaking on both LB Km and LB agar plates (Fig. 1E). These observations suggest that rapid plasmid curing in *P. putida* using a pBBR1MCS2-based CRISPR/Cas9 system is highly feasible, providing a practical tool for genome editing applications.

Various strategies have been reported for plasmid curing in *P. putida*. For instance, over 10 cycles of overnight culture in plain LB media have been used for plasmid curing in a three-plasmid genome editing system [14]. A self-curing pQURE vector has been shown to facilitate suicide plasmid-based genome editing and rapid plasmid curing in *P. putida* [30]. Additionally, the pFREE tool for rapid removal of various plasmids in *E. coli* and *P. putida* was developed by using CRISPR/Cas9 targeting of plasmid replicons [31]. Compared with these existing methods, our findings suggest a promising approach for more rapid and facile plasmid curing after genome editing, requiring only an 8 h culture without the need for additional plasmids or inducers.

### Application and optimization of CRISPR/Cas9 system

**Fig. 2:**
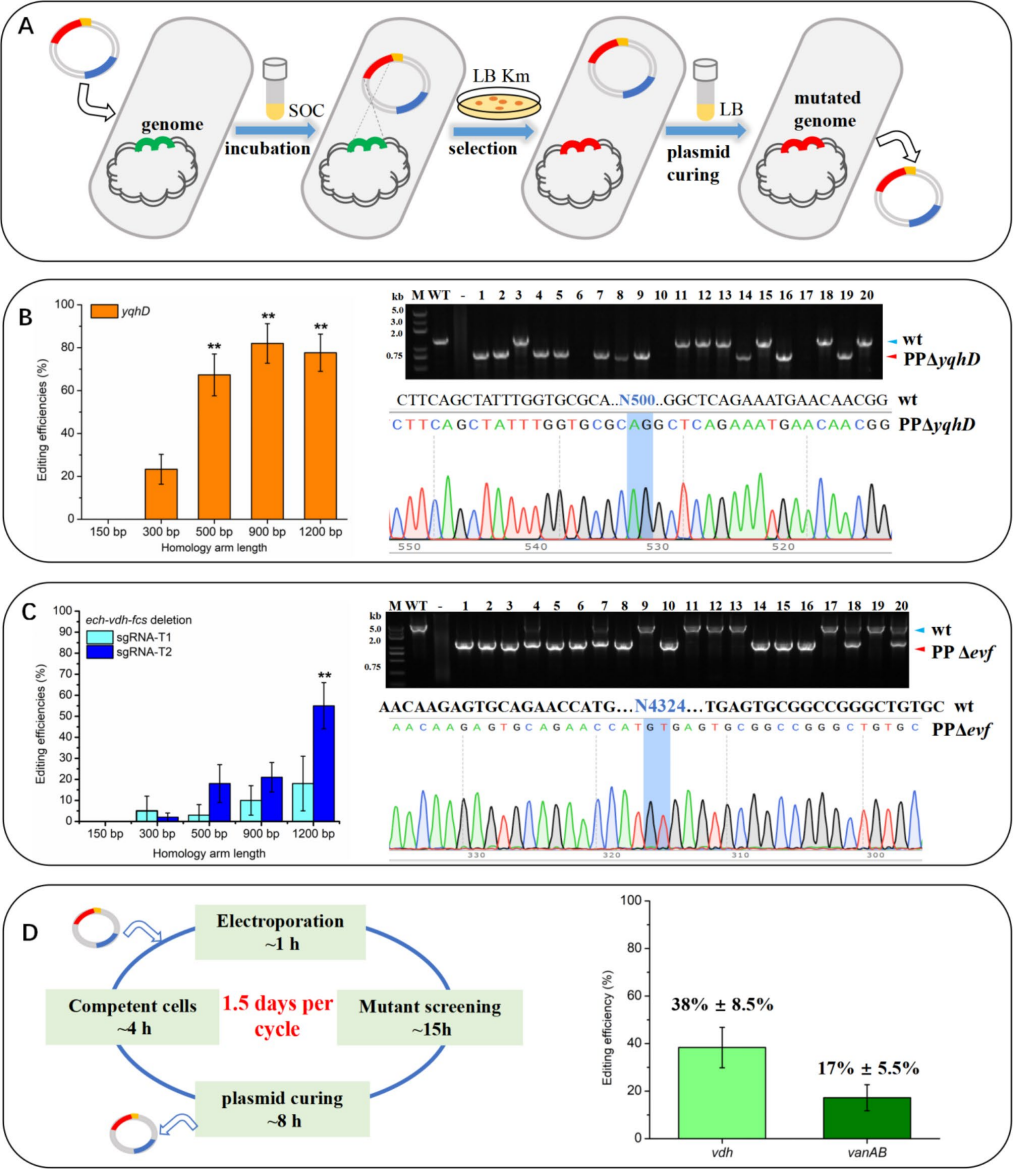
Application and optimization of the CRISPR/Cas9 system. (**A**) Schematic representation of using the “all-in-one” CRISPR/Cas9 system for genome edits in *P. putida* KT2440. (**B**) Efficiency of *yqhD* deletion with different homologous arm lengths and the editing verification by colony PCR and sequencing. (**C**) Efficiency of *ech-vdh-fcs* (*Δevf*) deletion using sgRNA-T1 andsgRNA-T2 with different homologous arm lengths, verified by colony PCR and sequencing. (**D**) Diagram of the iterative genome editing procedure and editing efficiency of sequential *vdh* and *vanAB* gene deletion. Statistical analysis was performed using one-way ANOVA, ** indicates *p* ≤ 0.01

Efficient and easy-to-operate genome editing using the CRISPR/Cas9 system is crucial for engineering *P. putida*, prompting us to construct and optimize a functional system in this study. The “all-in-one” CRISPR/Cas9 system (p3 in Fig. 1A) was constructed based on pBBR1MCS2, containing spCa9, sgRNA and homology arms, to facilitate streamlined genome editing and plasmid curing. Initially, we targeted a non-essential gene *yqhD* (PP_2492) with 5’-TCGGTAACCAAACCATTGCC-3’ as the target. The sgRNA, along with 1000 bp of the homology arms (500 bp each), was incorporated into pBBR-*Cas9* to generate pBBR-*Cas9ΔyqhD*-HA500. As illustrated in Fig. 2A, the CRISPR/Cas9 system for genome editing in *P. putida* utilizes an “all-in-one” plasmid introduced *via* a simple transformation process, recovered in SOC medium, and followed by colony selection and plasmid curing to enable precise and rapid genome modifications. Following this protocol, we optimized the deletion efficiency of the *yqhD* gene and the *ech-vdh-fcs* operon by varying the lengths of homologous arms and the selection of sgRNA targets.

Increasing the length of homology arms can enhance recombination efficiency by facilitating homology-dependent recombination [18, 32, 33]. The effectiveness of the CRISPR/Cas9 system for precise genome editing is influenced by factors such as Cas9 expression, sgRNA transcript levels, sgRNA target sites, and homology arm lengths. Although increasing sgRNA or Cas9 levels may enhance editing efficiency in systems using two or three plasmids [34], in our single-plasmid system, such strategies may lead to cellular toxicity or plasmid mutations, ultimately resulting in editing failure. Consequently, we opted to increase the length of the homology arms to optimize our editing outcomes. We engineered a series of plasmids with varying homology arm lengths, ranging from 150 bp to 1200 bp per arm, targeting deletions of *yqhD* (500 bp) and *ech-vdh-fcs* (4324 bp). As shown in Fig. 2B, we observed a significant improvement in *yqhD* deletion efficiency when the homology arm length increased from 150 bp to 500 bp, but there was no statistically significant difference when the length increased further to 1200 bp. The successful deletion was verified by colony PCR and sequencing. For the *ech-vdh-fcs* deletion depicted in Fig. 2C, increasing homologous arm length from 150 bp to 1200 bp improved editing efficiency from 3 to 20% when using sgRNA-T1, although the increase was not statistically significant. In contrast, with sgRNA-T2, editing efficiency significantly improved from 18% at 500 bp to 55% at 1200 bp. It is important to note that several colonies exhibited both wild-type and engineered band sizes, but these were not counted as positive edits. Based on these results and the costs of DNA fragment synthesis, we suggest using 500 bp homology arm length to ensure editing efficiency while facilitating synthesis and plasmid construction for future genome editing experiments. Theoretically, the CRISPR/Cas9 system we constructed allows a complete genome editing cycle to be finished in just 1.5 days. The rapid and straightforward plasmid-curing process facilitates subsequent rounds of genome editing using a new plasmid. As a proof of concept, we performed sequential genome edits to delete *vdh* and *vanAB*, achieving efficiencies of 38% for *vdh* (1440 bp) and 17% for *vanAB* (2025 bp), as shown in Fig. 2D.

Several CRISPR systems have been reported for genome editing in *P. putida*. For instance, a three-plasmid system consisting of pCas9 (a low-copy pRK2 plasmid with constitutive Cas9 expression and inducible λRed expression), pJOE (a suicide vector carrying the repair template), and pgRNA (pBBR1-UP carrying the sgRNA) was developed [12]. Their two-step protocol achieved a high gene deletion efficiency (85–100%). However, the protocol has several drawbacks: the chromosomal integration of pJOE is very inefficient, and the process requires two rounds of transformation and selection, which are time-consuming. Additionally, although pgRNA was easily cured while pCas9 was maintained, the co-transformation of pJOE and pgRNA for subsequent editing resulted in low editing efficiency. The ssDNA recombineering was merged with CRISPR/Cas9 in a three-plasmid system [14]. They achieved a large cluster (~ 69 kb) deletion; however, the complex system composition and time-consuming plasmid curing make it difficult to apply for iterative genome editing. A two-plasmid system was introduced into *P. putida*, with the pCas9 plasmid carrying both *Cas9* and *λRed* genes, and pSEVA-gRNA carrying the repair template and sgRNA [13]. They also incorporated *P*_*rhaB*_*-dgRNA* and *SacB* into the pCas9 plasmid, responsible for rhamnose-induced curing of pSEVA-gRNA and sucrose-assisted curing of pCas9, respectively. This system achieved 70–100% editing efficiencies and improved plasmid curing. However, the large size of the plasmid pCas9 reduced the electroporation efficiency. Additionally, the use of L-arabinose for λRed induction, rhamnose for P_rhaB_-dgRNA induction, and sucrose for *SacB* selection increased the operational complexity, requiring proficient skills to successfully carry out genome editing using this system. A one-plasmid system was constructed using a suicide plasmid pEMG carrying ThermoCas9/sgRNA/repair template, however, the low efficiency of chromosomal integration of pEMG is very inefficient and it requires two rounds of transformation and selection [16]. Another one-plasmid system was reported that integrated Cas9n/λRed cassettes into *P. putida* genome, which requires inducing and curing processes and extends the time needed to achieve a single gene deletion to at least four days [15]. Even though these methods reported high editing efficiency, the scientific community favored conventional methods over these CRISPR systems for *P. putida*, possibly due to multiple functional parts and complicated procedures, which hindered its adoption [5].

**Table 2:** Summary of gene deletions (9), substitutions (3), and insertions (2) conducted in this study

Editing Type	Gene location and function	Target size	Efficiency
**Deletions**			
*catBC*	PP_3715, PP_3714, Muconate cycloisomerase	1434 bp	13% (2/15)
*yqhD*	PP_2492, Iron-containing alcohol dehydrogenase	500 bp	59% (10/17)
*ech-vdh-fcs*	PP_3358, PP_3357, PP_3356, Enoyl-CoA-hydratase/aldolase, vanillin dehydrogenase, feruloyl-CoA-synthetase	4324 bp	50% (10/20)
*vdh*	PP_3357, Vanillin dehydrogenase	1443 bp	35% (7/20)
*vanAB*	PP_3736, PP_3737, vanillate-O-demethylase	2025 bp	17% (2/12)
*endA*	PP_3375, endonuclease I	960 bp	20% (4/20)
*endX*	PP_2451, Extracellular DNA endonuclease	687 bp	18% (2/11)
*phaG*	PP_1408, (R)-3-hydroxydecanoyl-ACP: CoA transacylase	882 bp	17% (3/18)
*phaC1ZC2*	PP_5003- PP_5005, poly(3-hydroxyalkanoate) polymerase 1, Poly(3-hydroxyalkanoate) depolymerase, Poly(3-hydroxyalkanoate) polymerase 2	4320 bp	83% (15/18)
**Substitutions**			
*yqhD::gfp*	PP_2492, Iron-containing alcohol dehydrogenase	500 bp::910 bp	14% (2/14)
*yqhD::rfp*	PP_2492, Iron-containing alcohol dehydrogenase	500 bp::714 bp	60% (12/20)
*PP_2827::MluI*	PP_2827, Zinc-dependent alcohol dehydrogenase	1080 bp::6 bp	13% (2/15)
**Insertions**			
::RBS *FabH*	Before PP_4379, Ketoacyl-ACP synthase III	39 bp	45% (9/20)
_*linker*_ *VS*	Behind PP_0528, Farnesyl diphosphate synthase	1812 bp	25% (5/20)

To assess the adaptability and user-friendliness of our single-plasmid CRISPR/Cas9 system, we conducted genome editing tasks with researchers of varying experience levels. An experienced PhD student performed a range of editing tasks, while two Master’s students, new to this technology, carried out the *catBC* deletion and *yqhD::gfp* substitution to assess the system’s ease of use. In total, 9 deletions, 3 substitutions, and 2 insertions were successfully conducted, as summarized in Table 2. The editing efficiency ranged from 13 to 83%, with an average of 37% achieved by the experienced PhD student. In contrast, the editing efficiency achieved by the two Master’s students was 13% and 14%, respectively. Despite these variations, the method demonstrated relatively good overall editing efficiency and usability.

These results effectively demonstrate the varying efficiencies of different gene editing using our CRISPR/Cas9 system in *P. putida* KT2440. Compared with existing CRISPR genome editing methods, our editing efficiency is not exceptionally high, and the deletion size is limited. However, the system offers significant advantages such as simple single-plasmid construction and a rapid gene editing and plasmid curing process. The successful experiments conducted by graduate students highlight the accessibility and efficacy of our CRISPR/Cas9 system, emphasizing its user-friendliness, even for novice users. These achievements illustrate the potential for widespread adoption of this single-plasmid-based, easily curable CRISPR/Cas9 system and underscore its practical applicability across various genetic research settings.

### Biosynthesis of valencene

As a demonstration of practical applications, we have engineered *P. putida* using the developed CRISPR/Cas9 system to achieve the biosynthesis of valencene. Valencene, prized for its citrus aroma, has been pursued for *de novo* synthesis in recombinant strains such as *S. cerevisiae* and *E. coli via* different pathways (Supplementary Fig. S6). *P. putida* KT2440 has emerged as a promising alternative due to its high tolerance to toxic byproducts, facilitating robust production processes for valencene and other terpenes.

**Fig. 3:**
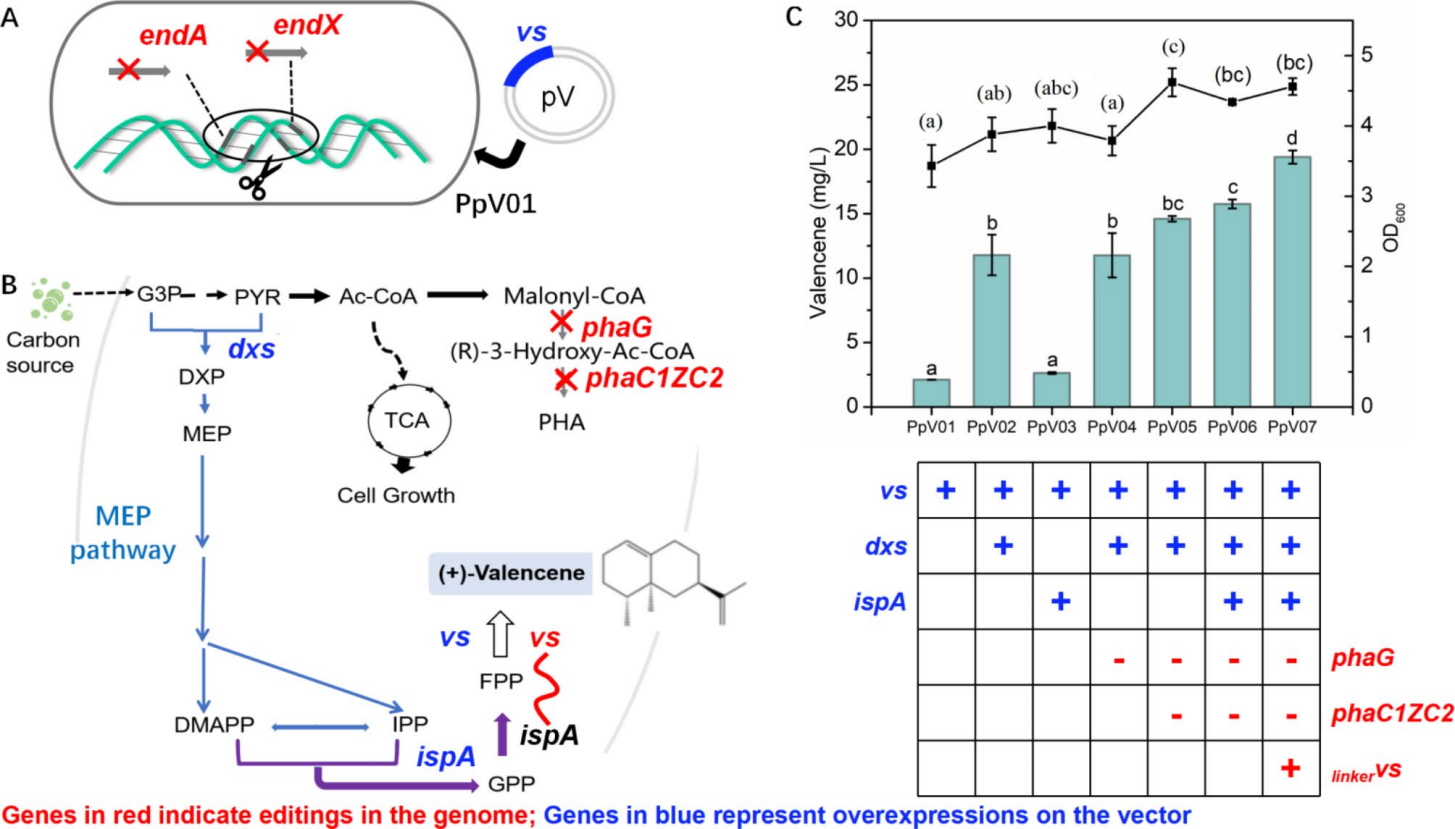
Iterative genome engineering of *P. putida* KT2440 for valencene production. (**A**) Illustration of the host strain *P. putida*. The *endA and endX* genes were deleted from the wild-type *P. putida* KT2440 strain to enhance the stability of exogenous gene expression. (**B**) Biosynthetic pathway of valencene in this study. Key engineering modifications are denoted by deletions in the genome (highlighted in red) and overexpressions on the vector (highlighted in blue). (**C**) Production of valencene utilizing engineered *P. putida* KT2440 in shake flasks. The symbol “+” indicates overexpression, while “-” represents deletion. The _*linker*_*VS* means that another copy of vs. gene was linked with *ispA* gene in the genome using a designed α-helical rigid linker (EAAAK)_2_. Abbreviations of chemicals and pathways: G-3-P, glyceraldehyde-3-phosphate; PYR, Pyruvate; Ac-CoA, acetyl-CoA; DXP, 1-deoxy-D-xylulose-5-phosphate; MEP, methylerythritol phosphate; DMAPP, dimethylallyl diphosphate; IPP, isopentenyl diphosphate; GPP, geranyl pyrophosphate; FPP, farnesyl pyrophosphate; PHA, polyhydroxyalkanoate; TCA, tricarboxylic acid cycle. Abbreviations of enzymes: vs., valencene synthase; *dxs*, DXP synthase; *ispA*, GPP/FPP synthase; *phaC1*, 3-ketothiolase; *phaZ*, acetoacetyl-CoA reductase; *phaC2*, PHA synthase; *phaG*, acyl transferase. The statistical significance among groups was denoted using letters: a, b, and c for valencene production, and (**a**), (**b**), and (**c**) for OD_600_. Variables sharing the same letter indicate no statistically significant difference between their means, while variables with different letters are significantly different (*p* < 0.05)

As shown in Fig. 3A, the *endA* and *endX* genes were deleted sequentially to create a host strain PpΔ*end*, enhancing exogenous gene expression stability [35]. Then, the plasmid pV01, carrying the valencene synthase (vs.) gene from *Callitropsis nootkatensis* [36], was introduced into PpΔend, resulting in the strain PpV01. This strain generated 2.11 mg/L of valencene. DXP synthase (*dxs*) is crucial in isoprenoid biosynthesis, playing a central role in the production of isopentenyl pyrophosphate (IPP) (Supplementary Fig. S6 and Fig. 3B). The IspA (GPP/FPP synthase) catalyzes the condensation of IPP and DMAPP to form farnesyl pyrophosphate (FPP), the valencene precursor. Overexpression *dxs* and *ispA* in PpV01 generated strains PpV02 and PpV03, respectively. Notably, PpV02 with *dxs* overexpression with VS showed a significant 4.5-fold improvement in valencene production, but overexpressing *ispA* with vs. in PpV03 did not increase production significantly. These results indicated that enhancement of key enzymes of the upstream pathway of MEP is favorable to increasing the yield of the target product mainly due to the insufficient supply of precursors of the upstream pathway at this time.

In *P. putida* KT2440, Ac-CoA can naturally be converted to polyhydroxyalkanoate (PHA) by *phaG* and *phaC1ZC2* [37]. Deleting *phaG* and *phaC1ZC2* genes may reduce precursor depletion by the bypass pathway thus improving valencene production. As shown in Fig. 3C, the *phaG* deletion in PpV02, that generated strain PpV04, did not improve valencene production, indicating the presence of other isoenzymes. However, further deletion of *phaC1ZC2* in PpV04, that generated strain PpV05, exhibited an 11.8% increase in yield compared to PpV02. Further overexpression of *ispA* in strain PpV06 significantly increased valencene yield and cell density compared to PpV04.

Finally, adding another copy of the vs. gene linked with *ispA* in genome using a designed α-helical rigid linker (EAAAK)_2_ [38] resulted in strain PpV07, which showed a significant improvement in valencene yield. Remarkably, the engineered PpV07 achieved a 10-fold increase in valencene production compared to the starting strain PpV01. Overexpressing *dxs*, *ispA*, and vs. genes mainly contributed to valencene yield, while deleting *phaG* and *phaC1ZC2* genes contributed indirectly by enhancing cell growth.

Heterologous biosynthesis of valencene has been reported in different microorganisms [39]. The highest valencene yield (12.4 g/L) was reported in *Saccharomyces cerevisiae* through gene screening, protein engineering, and pathway optimization [40]. Bacteria as hosts showed relatively lower valencene production, ranging from 2.41 mg/L in *Corynebacterium glutamicum* to 352 mg/L in *Rhodobacter sphaeroides* [39]. This study is the first report of valencene biosynthesis using *P. putida* as a host. Although our current yield is not as high as in engineered yeast, the fast growth rate of *P. putida* and our advantageous CRISPR/Cas9 editing technology allow for faster metabolic engineering, thus having great potential in increasing valencene production.

## Conclusion

Our study unveiled the easily curable nature of the pBBR plasmid carrying the CRISPR/Cas9 system in *P. putida* KT2440 without antibiotic pressure, leading to the development of a rapid, curable, and single plasmid-based CRISPR/Cas9 system. By prioritizing simplicity and ease of use, this system offers a straightforward approach for researchers with varying levels of experience in genomic editing, allowing one editing in less than 1.5 days and facilitating iterative genome edits. This practicality opens opportunities for diverse applications, emphasizing the system’s versatility.

By streamlining the genetic manipulation process, we successfully achieved the first biosynthesis of valencene with an impressive 10-fold increase in production using *P. putida* KT2440, showcasing the system’s potential for expanding biotechnological applications. Our laboratory is also interested in utilizing this system to knock out genes involved in biofilm formation in *P. putida* to identify key genes that enhance bacterial accessibility to plastic surfaces and improve plastic degradation. Additionally, we are exploring the use of this CRISPR/Cas9 system to investigate the spatial localization of key membrane proteins, such as deletions, insertions, or fluorescent tagging of specific proteins, thereby advancing our understanding of cellular signaling and metabolic pathways. The system is now available on Addgene (NO 220369) for global distribution, underscoring its value to the broader scientific community.

## Electronic supplementary material

Below is the link to the electronic supplementary material.

Supplementary Material 1

## Data Availability

No datasets were generated or analysed during the current study.

## Abbreviations

CRISPR: Clustered Regularly Interspaced Short Palindromic Repeats
LB: Luria–Bertani
SOC: Super Optimal Broth with Catabolite repression medium
Km: Kanamycin
Cm: Chloramphenicol
PCR: Polymerase Chain Reaction
sgRNA: Single guide RNA
PAM: Protospacer adjacent motif
G-3-P: Glyceraldehyde-3-phosphate
PYR: Pyruvate
Ac-CoA: Acetyl-CoA
DXP: 1-deoxy-D-xylulose-5-phosphate
MEP: Methylerythritol phosphate
DMAPP: Dimethylallyl diphosphate
IPP: Isopentenyl diphosphate
GPP: Geranyl pyrophosphate
FPP: Farnesyl pyrophosphate
PHA: Polyhydroxyalkanoate
TCA: Tricarboxylic acid cycle
vs: Valencene synthase
dxs: DXP synthase
ispA: GPP/FPP synthase
phaC1: 3-ketothiolase
phaZ: Acetoacetyl-CoA reductase
phaC2: PHA synthase
phaG: Acyl transferase
