# Supplementary material for: A single-plasmid-based, easily curable CRISPR/Cas9 system for rapid, iterative genome editing in Pseudomonas putida KT2440
Source: Microb Cell Fact. 2024 Dec 30;23:349. doi: 10.1186/s12934-024-02634-4 (PMC11684315; doi:10.1186/s12934-024-02634-4)
Supplement: Supplementary file 1 — Supplementary Material 1 [file 12934_2024_2634_MOESM1_ESM.docx]

Supplementary files for

A single-plasmid-based, easily curable CRISPR/Cas9 system for rapid, iterative genome editing in Pseudomonas putida KT2440

Qifeng Wen ^1, 2^, JinJin Chen ^3^, Jin Li ^3^, Ida Putu Wiweka Dharmasiddhi ^3^, Maohua Yang ^1,^ *, Jianmin Xing ^1, 2,^ *, and Yilan Liu ^3,^ *

^1^ State Key Laboratory of Petroleum Molecular & Process Engineering, Institute of Process Engineering, Chinese Academy of Sciences, Beijing, China

^2^ College of Chemical Engineering, University of Chinese Academy of Sciences, Beijing, China

^3^ Department of Chemical Engineering, University of Waterloo, Waterloo, Canada

* Corresponding authors:

Maohua Yang, Email: mhyang@ipe.ac.cn

Jianmin Xing, Email: [jmxing@ipe.ac.cn](mailto:jmxing@ipe.ac.cn)

Yilan Liu, Email: yilan.liu@uwaterloo.ca

The file includes:

Supplementary Table S1-S2

Supplementary Figures S1-S6

Table S1 Primers used in this experiment.

| Primers | Sequences |
| --- | --- |
| IR9s6 | aaccaatacgtgaacaagcagaa |
| IR9s7 | AAACGGAGGAATGGGAACG |
| tGyqhdF2 | TGGCGAATCTGCGTCTGC |
| tGyqhdR2 | GAGTGTCCGGGTCTGTTGTATG |
| tppyqhdF | TGCCTAGCAACAACCCTCG |
| tppyqhdR | AGCATCAGCGGACCGATTT |
| t2287F | GGTCTACACGACGCAGGTCG |
| t2287R2 | CACGCAACATGGCCAGCAGC |
| tGrbsfabHF | CCAAAAAATTTTTAAATAAGGAGGCTG |
| tGrbsfabHR2 | GGATGCAGAAGGCGTCGATATC |
| tGyqhdR3 | CGCCGCAACAGGCTTGCCGGCG |
| vdhsRNAF | ctagtAACCTTCGAGCGCTGTAACCgttttagagctagaaatagcaagttaaaataag |
| vdhgRNAR | GTTGATTTCCGACAGGCCGAAGGTCGgcttctgcagCATTATACG |
| vdhupF | CGACCTTCGGCCTGTCGGAAATCAACTGGGGCATCCCGCCTGGTAAC |
| vdhupR | GGCCTGCCGCGGCTACATGCTCATTCCTCTTGTTGTCGTTATAG |
| vdhdownF | CAAGAGGAATGAGCATGTAGCCGCGGCAGGCCCAGACCTCCGGC |
| vdhdownR | CCAGACTATACTAGACTGCAGACACCGGGCAATAGGCAATACCGGC |
| GvdhF | CTCGACGACAAGAGCATCAAG |
| GvdhR2 | GAACGGCTGGCTGTCGCTGA |
| pCas9vdhF | GTGTCTGCAGTCTAGTATAGTCTGGAACAGCGCACTTACGGGTTGCTGCG |
| pCas9vdhR | cGGTTACAGCGCTCGAAGGTTactagtattatacctaggactgagctagctg |
| vanABsRNAF | ctagtGTGAGTGATGAGTGGGCCTAgttttagagctagaaatagcaagttaaaataag |
| vanABsRNAR | GATCGACGACGTTCCGCTCGATGGACGTgcttctgcagCATTATAC |
| vanABupF | gaagcACGTCCATCGAGCGGAACGTCGTCGATCAGAATACGGCC |
| vanABupR | GTCGTAAGACGGGTCACATGGGAGGCTCTCCGGGTTTTG |
| vanABdownF | GAGCCTCCCATGTGACCCGTCTTACGACGAGGGCAGGATGAC |
| vanABdownR | CCAGACTATACTAGACTGCAGCGGGTGCGGTCGAGGTGCGCGGTGTGC |
| pCas9vanABF | GCTGCAGTCTAGTATAGTCTGGAACAGCGCACTTACGGGTTGCTG |
| pCas9vanABR | TAGGCCCACTCATCACTCACactagtattatacctaggactgagctagctgtcaaG |
| GvanABF | CATTGACCTACCACGCCGAC |
| tGrbsfabHF | CCAAAAAATTTTTAAATAAGGAGGCTG |
| DHA150F | ctctagagtcgacctgcagaagcTGCCTAGCAACAACCCTCGACATCG |
| DHA150R | CCAGACTATACTAGACTGCAGTACGCCGTTTGCGAGTTGGCGGGTAG |
| DHA300F | ctctagagtcgacctgcagaagcTGCATCAGTCGATGCGCTGCCAATG |
| DHA300R | CAGACTATACTAGACTGCAGGGCGCGGGTGCTGTAGTCCGCAGAC |
| yqhDup900F | tctagagtcgacctgcagaagcCATTCGAGGACAGCGAGGAGGATG |
| yqhD900downR | GTTCCAGACTATACTAGACTGCAGAGCTCTTCGAGCCAGAAACCGGTC |
| yqhD1200upF | tctagagtcgacctgcagaagcGCTTCGTGTTCCGCACCACGGGC |
| yqhD1200downR | GTTCCAGACTATACTAGACTGCAGTTGCCGGCGGCGATGGAGCGCTCA |
| speIevfgRNA2F | CTAGactagtCAGCGACTGACGATGGCCAAgttttagagctagaaatagc |
| evfSgRNAsalIR | ACGCgtcgactctagagaattc |
| evfupF | ctctagagtcgacctgcagaagcACCGCACGCGCACGGCCAGCGCGCG |
| evfupR | GCACAGCCCGGCCGCACTCACATGGTTCTGCACTCTTGTTGTTCGAGG |
| evfdownF | AGTGCAGAACCATGTGAGTGCGGCCGGGCTGTGCACCGTC |
| evfdownR | CAGACTATACTAGACTGCAGGATCATGTCCACGCCGGCAGCCGGGTCG |
| evf150upF | attctctagagtcgacctgcagaagcTCCTTATGCGATTCGGCTAGAGAG |
| evf150downR | GTTCCAGACTATACTAGACTGCAGCTGCACGTGCCAGCACCGCGCG |
| evf1200upF | ctctagagtcgacctgcagaagcCAGGCTGAAAGGGTTGCCAGTG |
| evf1200downR | CAGACTATACTAGACTGCAGCTTGGCCACCGCCGATGCATGCCG |
| evfup300F | ctctagagtcgacctgcagaagcGCGCGCGTCGCAAGGCGTAGCCGATC |
| evfdown300R | CCAGACTATACTAGACTGCAGCCGTGCCACAGATGCGCTGCACCGCC |
| evf900upF | ctctagagtcgacctgcagaagcATCAGGAAACTGATGGCGGCACG |
| evf900downR | CAGACTATACTAGACTGCAGGCACGCCAACAGCCGTGGCCATCAG |
| GevfF | GCAGCGCGCCTAACCCCAGG |
| GevFR3 | GAGGTCGACGTCGGCATATG |
| dCatBC-F | cactggctgacacgaatgct |
| dCatBC-R | caggcaaaatcacagtgacataac |
| endAupF | ctctagagtcgacctgcagaagcCGCAGCACGGGGGTTGAGCATTAC |
| endAupR | CCTTTGAAACGGTCACATGGGGAAAACATATTTCAGGTTG |
| endAdownF | TATGTTTTCCCCATGTGACCGTTTCAAAGGCTGCGCGGCCCCTG |
| endA downR | CAGACTATACTAGACTGCAGTGCGCGTGCTGTATCACAGCCAGCG |
| endASgRNASpeIF | CTAGactagtCGCATCGACGCTGCCTCGTGgttttagagctagaaatagc |
| GendAF | CATGCTCGACTCAGCCACATTC |
| GendAR | GGCAGTGATGTGCATGGCAAG |
| endXSgRNAspeIF | CTAGactagtCCTCGCCTTGAAGTCGACCTgttttagagctagaaatagc |
| endXupF | ctctagagtcgacctgcagaagcAAAGAGCTGCAGCGGATCTTCTG |
| endX upR | CTGGCCTGAGGATCACATGCGCAGTCAATCTTCCTTCGGT |
| endXdownF | GATTGACTGCGCATGTGATCCTCAGGCCAGCGTTTGTAAGTAGGC |
| endXdownR | CAGACTATACTAGACTGCAGTGTGTCAGGCGCCGTTCACACAAG |
| GendXF | GTACGCATCCAGAAGATCATC |
| GendXR2 | CCGATTCGGTGGTCATCCAG |
| phaGSgRNAspeIF | CTAGactagtTGGACTTGCCTGAATACGGCgttttagagctagaaatagc |
| phaGupF | ctctagagtcgacctgcagaagcCGATCGTCGAGGGGCGTTACCACCAG |
| phaGupR | CGCCGAGCCGCTCACATGTCATCGACTCCTGGCG |
| phaGdownF | GTCGATGACATGTGAGCGGCTCGGCGCCTTGTAG |
| phaGdownR | CAGACTATACTAGACTGCAGGATCGCGGCTACCGACAGGCCCGCTTG |
| GphaGF | CTGCATGTGACTACCAAGTC |
| GphaGR | GACCATCATCGGTAGCACAC |
| phaABCgRNAspeIF | CTAGactagtGTACAGCCCCATCACCGAGCgttttagagctagaaatagc |
| phaABCupF | ctctagagtcgacctgcagaagcCGAGCTGCAAGGCCGCCTGCCGATTCG |
| phaABCupR | CCAGTCAGCAGCTCACATCTACGACGCTCCGTTGTCCTGAGAC |
| phaABCdownF | GCGTCGTAGATGTGAGCTGCTGACTGGATGAAAACCCGCGATC |
| phaABCdownR | CCAGACTATACTAGACTGCAGAGCCGCAACGAGATCAAGGCACTGC |
| GphaABCF | CATCTGTTTGAATGACTTGC |
| GphaABCR | GCCAAGTCTTCGACCACTTC |
| ispAvsSgRNAspeIF | CTAGactagtTAGGTGAAGCTGTTTAGTTAgttttagagctagaaatagcaag |
| ispAvsup900F | ctctagagtcgacctgcagaagcCGCGGAGCCGGAAGCATGATTGGCA |
| ispAvsupR | CATCTCGGCCTTGGCTGCCGCTTCGTTACGGCGTTCGACAATATAAC |
| linkerVSF | GAAGCGGCAGCCAAGGCCGAGATGTTCAACGGCAACAG |
| linkerVSdeR | CAGGGATCGGtgaCGCGGTTCAGTGATGGTGATGGTGGTGCG |
| ispAvsdowndeF | CCATCACTGAACCGCGTCACCGATCCCTGTAGGATCGGGTTTACCCGCGAATGCGATC |
| ispAvsdown900R | GTTCCAGACTATACTAGACTGCAGCTTGGCGTATTCCTCGGTGCGACGGGC |
| GispAvsF | CCTATGCAATCGAACTGCGC |
| GispAvs900R2 | CAGGTCACGCATGTTGCGCAG |
| pBBRCnVS(rbsdxs)F | cgcggtggcggccgctctagaTAAGGAGGTCTTTTTATGCCCACGACGTTTCAAGAG |
| pBBRCnVS(rbsdxs)R | agcccgggggatccactagtCTAGAGCCCGAGCCTGGCCATG |
| DXSispAF | GGCTCGGGCTCTAGactagtTAAGGAGGACGGGTTATGATTGGCAC |
| DXSispAR | gcccgggggatccactagtTTAGTTACGGCGTTCGACAATATAAC |

Table S2 Spacer sequences of targeted genes in this study.

| Targeted genes | Spacer sequences | PAM |
| --- | --- | --- |
| *yqhD* deletion | TCGGTAACCAAACCATTGCC | CGG |
| *vdh* deletion | AACCTTCGAGCGCTGTAACC | CGG |
| *vanAB* deletion | GTGAGTGATGAGTGGGCCTA | CGG |
| *catBC* deletion | AGCGCCTTGCTCGACGCCCA | GGG |
| *endA* deletion | CGCATCGACGCTGCCTCGTG | TGG |
| *endX* deletion | CCTCGCCTTGAAGTCGACCT | GGG |
| *phaG* deletion | TGGACTTGCCTGAATACGGC | TGG |
| *phaC1ZC2* deletion | GTACAGCCCCATCACCGAGC | AGG |
| *RBS FabH* insertion | CATTGGGAAGCCAGAATGAT | TGG |
| *yqhD::rfp* substitution | TCGGTAACCAAACCATTGCC | CGG |
| *yqhD::gfp* substitution | TCGGTAACCAAACCATTGCC | CGG |
| *PP_2827::MluI* substitution | CAAGACCTGCTGCTGCAGAT | TGG |
| *_linker_VS* insertion | TAGGTGAAGCTGTTTAGTTA | CGG |


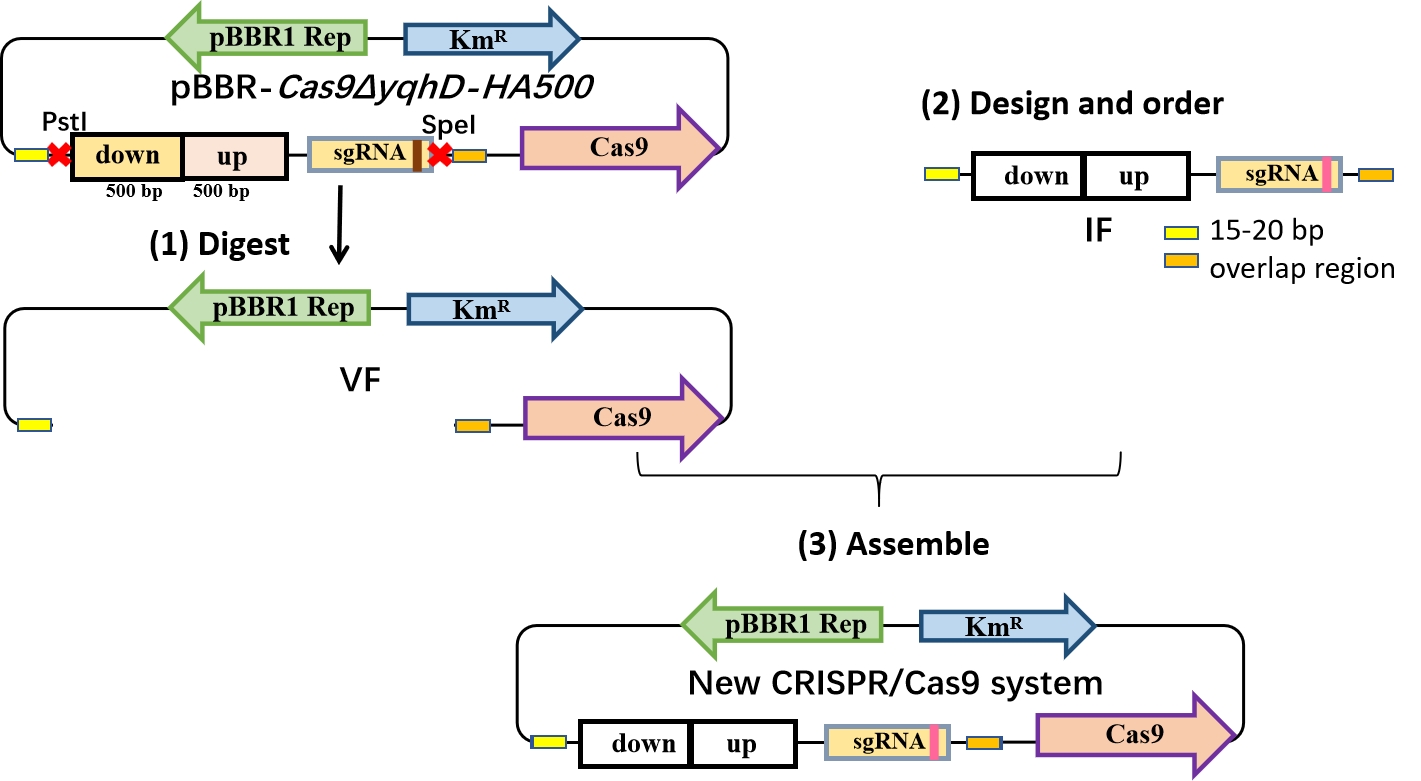


Fig. S1 Construction of the CRISPR/Cas9 for new target. (1) Digest pBBR-Cas9ΔyqhD-HA500 with PstI and SpeI to obtain the vector fragment (VF); (2) Design and order the insert fragment (IF) for the new target gene based on the sgRNA design described in Section 2.2; (3) Assemble the VF and IF into a new CRISPR/Cas9 system for genome editing.


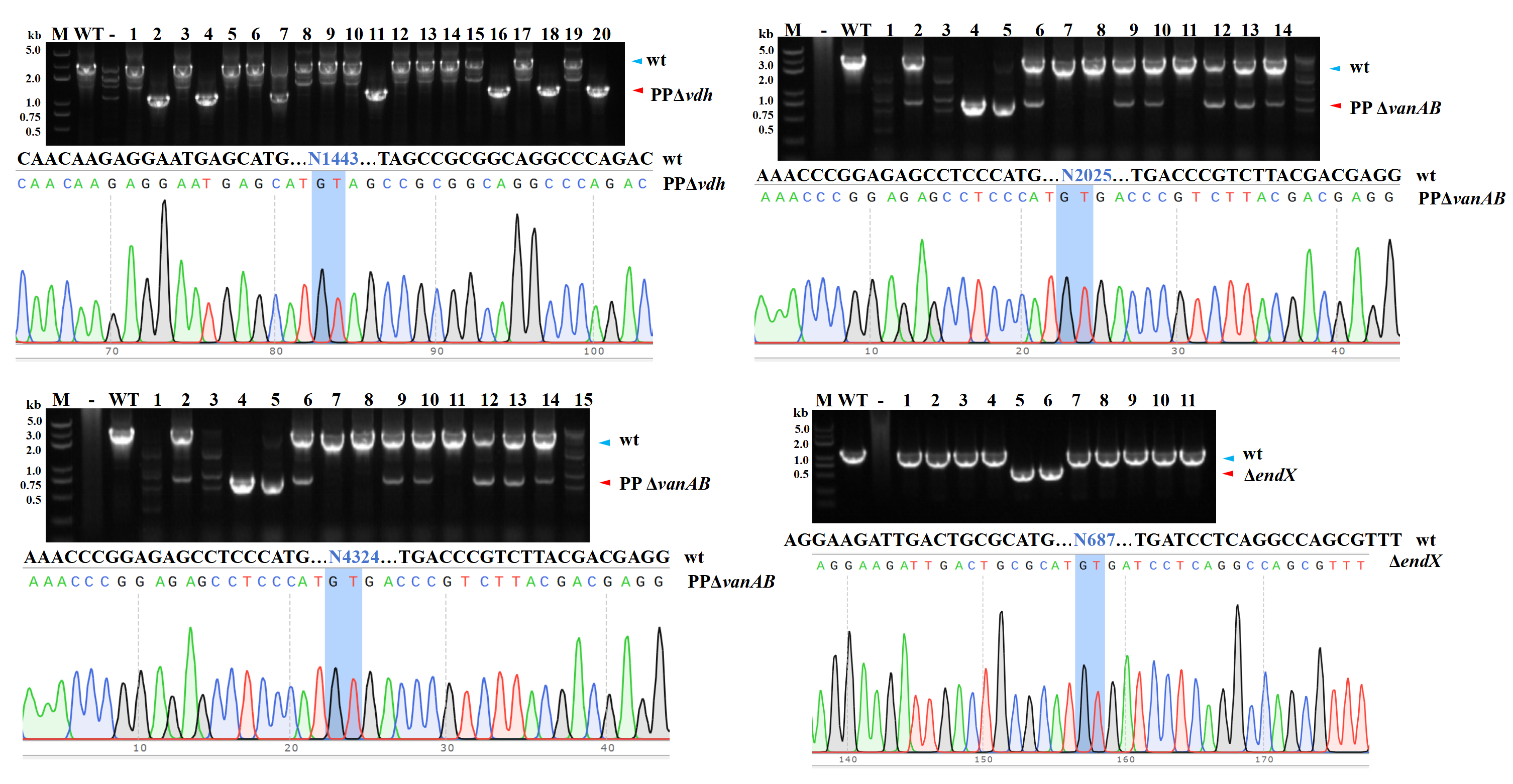


**Fig. S2** PCR verification and sequencing of all genome editing in *P. putida* KT2440 in this study. To be continued on next page.


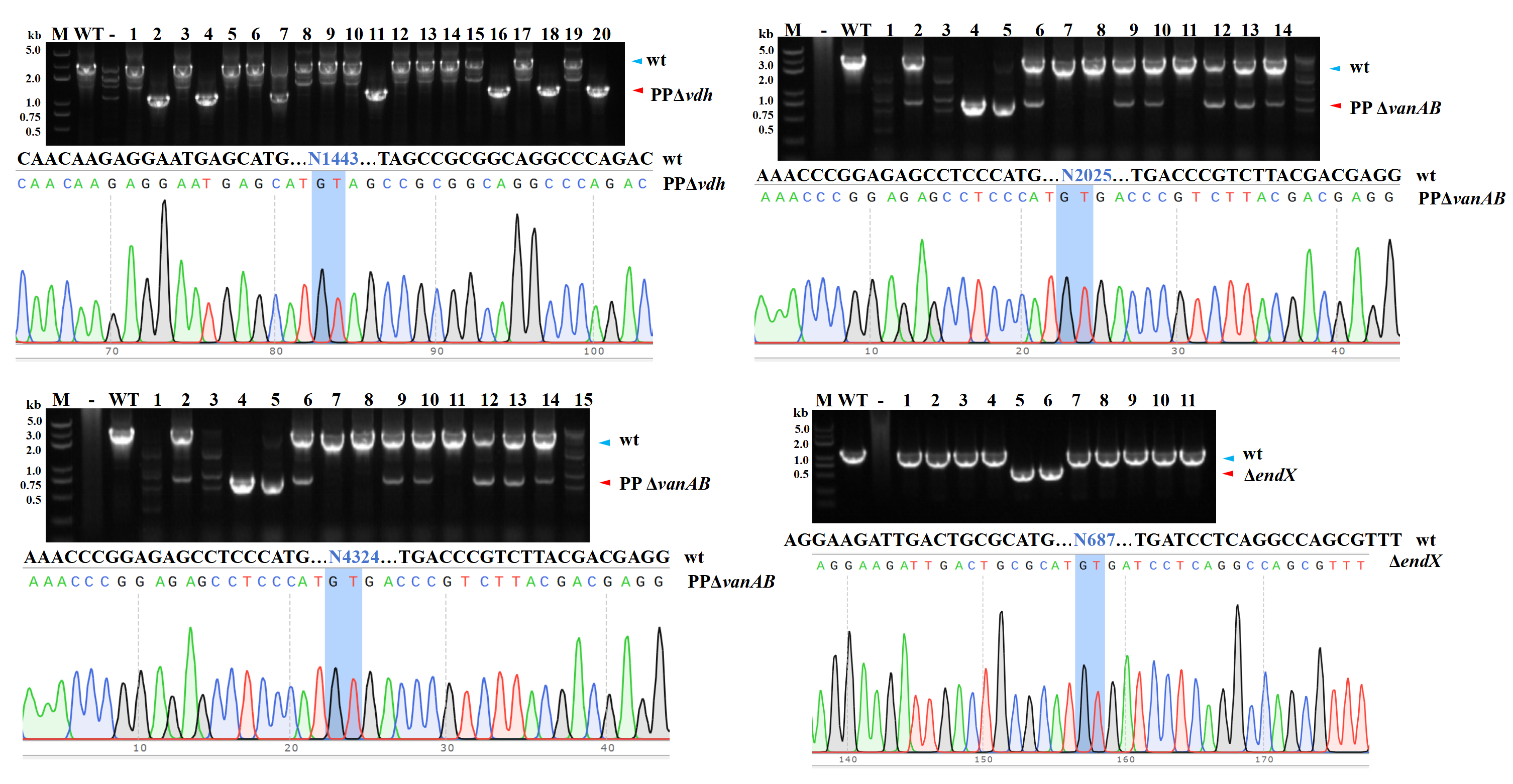


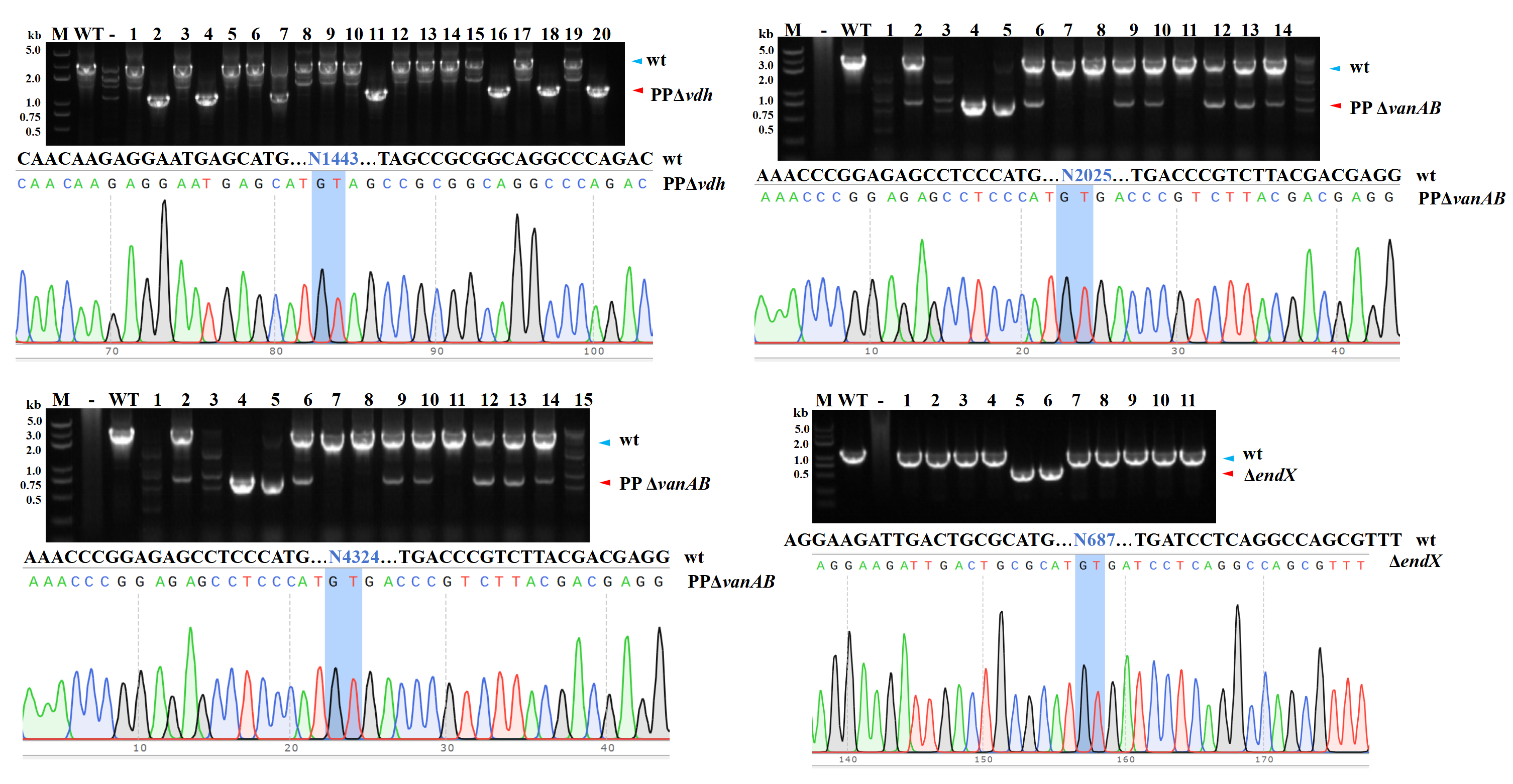


Continued **Fig. S2** PCR verification and sequencing of all genome editing in *P. putida* KT2440 in this study.


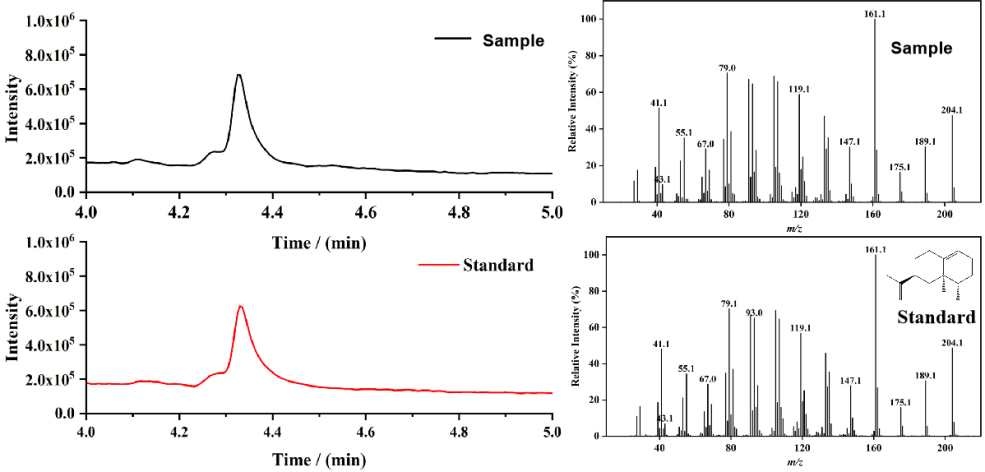


Fig. S3 GC-MS analysis of retention time and mass fragmentation for both a sample and a standard of valencene.


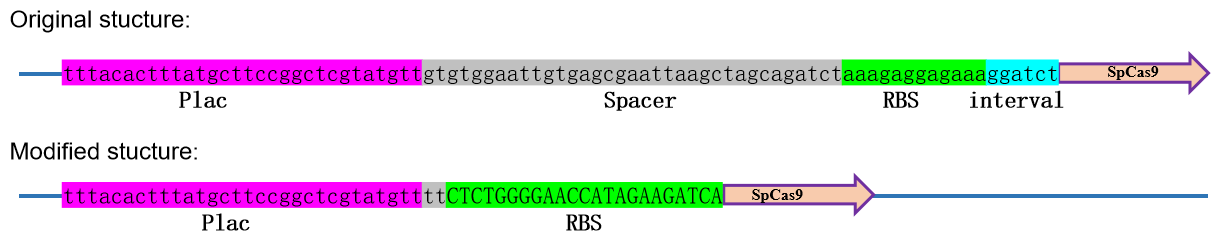


Fig. S4 Modified sequence before spCas9 to reduce its expression level. The spacer and interval were removed and the RBS was changed.


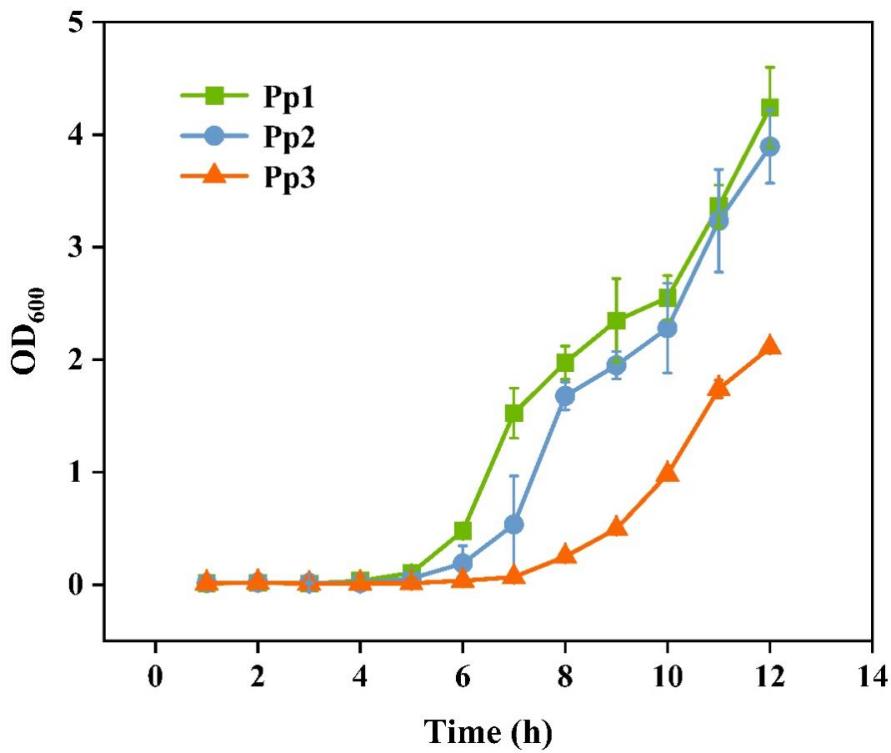


**Fig. S5** Growth curves for the Figure 1 strains in the presence of the antibiotic kanamycin (Km). Pp1, green, Pp2,blue; Pp3,orange. Cells were grown in LB at 30 ^o^C, and three independent replicates were conducted for each strains.


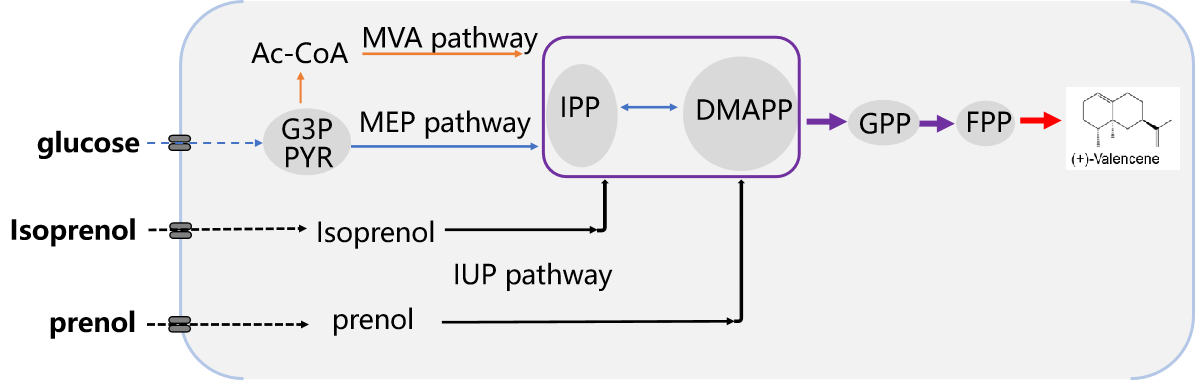


Fig. S6 Pathways for valencene synthesis. The valencene synthase (vs) was heterologously expressed to catalyze the formation of (+)-valencene from Farnesyl diphosphate (FPP) and geranyl diphosphate (GPP). These precursors typically originate from the condensation of dimethylallyl diphosphate (DMAPP) and isopentenyl diphosphate (IPP), which are synthesized via the methylerythritol 4-phosphate (MEP) pathway, the mevalonate (MVA) pathway, and the isopentenol (IUP) pathway.
